# Between the Bazaar and the Bench: Making of the Drugs Trade in Colonial India, ca. 1900–1930

**DOI:** 10.1353/bhm.2016.0017

**Published:** 2016

**Authors:** Nandini Bhattacharya

**Keywords:** drugs trade, Ayurveda, bazaar medicine, adulteration, colonial India, medical market, Drugs Enquiry Committee, indigenous drugs

## Abstract

This article analyzes why adulteration became a key trope of the Indian drug market. Adulteration had a pervasive presence, being present in medical discourses, public opinion and debate, and the nationalist claim for government intervention. The article first situates the roots of adulteration in the composite nature of this market, which involved the availability of drugs of different potencies as well as the presence of multiple layers of manufacturers, agents, and distributors. It then shows that such a market witnessed the availability of drugs of diverse potency and strengths, which were understood as elements of adulteration in contemporary medical and official discourse. Although contemporary critics argued that the lack of government legislation and control allowed adulteration to sustain itself, this article establishes that the culture of the dispensation of drugs in India necessarily involved a multitude of manufacturer–retailers, bazaar traders, and medical professionals practicing a range of therapies.

At the turn of the nineteenth century colonial India was awash with patent and proprietary medicines, tinctures, tonics, powders, and tabloids of every description. Many were imported from Great Britain or the United States; a substantial number also arrived from Germany, France, Italy, and the fast-industrializing Japan. These were sold by British Indian agents in India, who also traded in compounds and galenicals that they manufactured themselves. They competed with Indian druggists, who were large-scale importers and also did extensive business with fledgling Indian firms in the early twentieth century. Therapeutic and cosmetic[Other P-61] products of varying standards and efficacy were sold directly to consumers by small merchants, itinerant traders, and Ayurvedic and Hakimi practitioners as well.

This article argues that the prevailing distinctions drawn between indigenous and Western drugs in colonial India are misleading. These distinctions are premised on an understanding of cultural nationalism. They do not take into account the heterogeneous nature of the trade, manufacture, and consumption patterns in the market. An understanding of this composite nature of the drug market provides a unique entry point to the question of adulteration of drugs within that market. The heterogeneity of the market did not only involve the availability and diversity of drugs or the multiple layers of agents and distributors involved in that trade, although these were characteristic of that market. This market witnessed the availability of drugs of diverse potency and strengths, sometimes one single drug sold in several degrees of potency. This was as true of Ayurvedic and Hakimi medicines as of Western therapeutic products. For the latter, there was always British Pharmacopeia (BP) as the standard of reference, but neither the importers not local manufacturers were restricted by its recommendations. This diversity of drugs and potencies was defined within contemporary medical, official, and public discourse as “adulteration.” This article proposes adulteration as the key trope of the colonial Indian drug market.

Adulteration of drugs in this market has been almost entirely ignored by historians, except with reference to the distribution of quinine. Patricia Barton has pointed out that the colonial government’s efforts to distribute cheap packets of quinine throughout India failed due to flaws in distribution and the common adulteration prevalent in the use of cinchona and its alkaloids in India.^1^ While Barton has made an important contribution to the history of adulteration of drugs, her perspective is limited to quinine. The problems of adulteration in quinine (or cinchona) cannot be seen in isolation to the entirety of the political and cultural complexities that defined the drug market in colonial India. This article explores the story of heterogeneity and adulteration in the emergent Indian drug market. It argues that the discourse of adulteration in drug production and marketing in India was informed by uniquely Indian realities, such as the share of the indigenous drugs in the market, economic nationalism (*swadeshi*), the elusive allure of import substitution, and a plurality of medical traditions and practices. The problem of adulteration of drugs in India is wider than that of a lack of government regulation. This article[Other P-62] explains how the drug market in colonial India was formed and why it remained largely uncontrolled in spite of state or nationalist intervention.

Historians of medicine have examined the histories of the pharmaceutical industries in Europe and North America, focusing on industrialization, collaborations between the industry and medical schools, and new marketing strategies in the late nineteenth and twentieth centuries.^2^ These studies have emphasized that organization, often into cartels (in the United Kingdom), and marketing strategies helped British drug companies to expand in the late nineteenth century, while the German industry largely depended on research and the production of new patents. In the United States, collaborations between universities and corporations helped in the manufacture of new drugs.^3^

Although scholarship in Indian history of medicine has explored how colonialism changed institutions and praxis of medicine in India, there is relatively scant attention to a history of the production and the delivery of drugs. Recent histories on drugs in India have focused on Ayurvedic and Unani therapy: their marketing, standardization, and tangled relationship with the British Indian medical establishment and with Indian nationalism. These narratives of the “modernization” and nationalization of indigenous drugs focus on the cultural nationalism, specifically in science and medicine through an interaction of their practitioners with several strands of Indian nationalist activity. This provided the political context of the consolidation of the corporate identities of Ayurvedic and Unani practitioners and informed new marketing strategies, transforming traditional drugs into modern Ayurveda or Unani therapeutics. Ayurvedas and Hakims used the print media in major Indian languages of Punjabi, Hindi, and Urdu to sustain the discourse of the Indian body, with nationalist political movements in order to build the edifice for a legitimate,[Other P-63] collective identity for themselves.^4^ The construction of this indigenous collective was not seamless; as Kavita Sivaramakrishnan has pointed out, the Punjabi *vaids* resisted the Hindi-dominated discourse that validated Hindu nationalism and instead opted to appropriate “indigenous” science in Punjabi language and a Sikh solidarity.^5^ In the Unani tradition, as Seema Alavi has argued, the discourse of authentic Unani practitioners in the Urdu public sphere occurred in counterpoint to the rise of the self-qualified hakim and the dissemination of Unani knowledge as well as its consolidation in print and practice in north India.^6^ In the princely court of Hyderabad, the practice of Unani bypassed the contentious debates between the reformers and traditional Unani practice was absorbed into an institutionalized hospital system.^7^

Rachel Berger has claimed that the Indigenous Drugs Committee, instituted by the government of India, privileged a few useful Indian drugs, but not the knowledge system of Ayurvedic medicine, which instead “presented medically useful components of Indian agriculture as something that Europeans had stumbled upon, of which Indians had been unaware.”^8^ This is inaccurate; on the contrary, BP incorporated fifty-odd Indian drugs in its colonial addendum as early as in 1899.^9^ Instead, it is possible to argue that while Ayurvedic drugs were incorporated within BP, their usage by Western practitioners was tolerated within limits; the “bazaar medicines,” drugs from the local markets, were regularly used even in hospitals and dispensaries in British India and were “in every way efficient substitutes for the better known drugs of BP.”^10^ Projit Mukharji has shown that both[Other P-64] Ayurveda and Hakimi in colonial India consolidated themselves through exclusions of subaltern knowledge and practice, and by borrowing the discourse of Western, scientific modernity.^11^ In a broader context, Mukharji has argued for the “vernacularization” and “provincialization” of Western medicine especially in Bengal as Bengali medical print in the nineteenth century was adapted to local medical vocabulary and praxis.^12^

Prior researchers have focused on Indian practitioners and the modernizing of indigenous drugs centered on the trope of indigenous medical cultures in consonance with Indian nationalism in various forms. Sivaramakrishnan, Berger, and Madhuri Sharma have specifically demonstrated that indigenous medical theory and praxis, particularly that of Ayurveda, were constructed through the public sphere in Punjabi and Hindi in northern India.^13^ They have demonstrated, valuably, the workings of medical science at the sites of Indian culture and politics and its interactions with Western medicine and colonial modernity. Their scholarship has provided a counterfoil to the focus by historians on the loss of institutional support and legitimacy of the indigenous medical systems in British India.^14^

To move from discourse to praxis, international trade in drugs and therapeutic products had burgeoned in the eighteenth century, commensurably with the volume of trade itself. With the isolation of the “active principles” of drugs from their raw materials, the potency of drugs for sale increased as well.^15^ As Guy Attewell has demonstrated, drugs traveled and were easily assimilated within different cultural traditions in the age of commerce, making them heterogeneous.^16^ Indeed, as anthropologists[Other P-65] have pointed out, even in the contemporary age of the dominance of biomedicine, medicines and therapeutic substances used by medical practitioners have remained heterogeneous, borrowed from several existing medical traditions.^17^ Discussions on indigenous or Indian medicine have therefore focused on the epistemic and discursive trajectories, with emphasis on cultural aspects of medicine, but have ignored the structure of the market itself. This essay asks some questions: Did nationalist and institutional consolidations change the content of medicines and therapeutic substances sold in colonial India? How were they distributed and marketed? Was the widespread adulteration of the market the consequence of its heterogeneity? To what extent did the involvement of government-sponsored medical establishments that bought therapeutic products from the market influence the drug market?

The drugs and therapeutics trade in India was a complex one and occurred at multiple levels. These processes of production, marketing and dispensing of Indian drugs were conducted in an intensely competitive, dynamic, and diverse market. The polarities of indigenous and Western therapeutic products were a construct of this market. The terms “Western” and “Indian,” “Ayurveda” and “Allopathy,” “Unani” and “Doc-tory,” and “swadeshi” and “foreign” had deep political resonance, but these dialectics hardly represented the totality of the drug market and were reflective, often, of marketing strategies. Instead, the production and consumption of drugs and other therapeutic products in the late nineteenth- and early twentieth-century India were ideologically flexible and commercially vibrant. An international, heterogeneous therapeutic culture placed India within this trade; and this transnational commerce encompassed and included regional therapeutic vernaculars, both in medical print and therapeutic products. This market was made mostly in Indian cities. And although the fear of adulteration was pervasive, it was not a construct of the heterogeneity of the market but an inalienable part of the making of the market itself.

## The Making of the Indian Drugs Trade

The drug market was formed through the interplay of three institutions: the colonial state, the emergence of the Indian middle class, and the drugs trade. These defined the heterogeneity of the market and led to various degrees of adulteration in the drug market.[Other P-66]

The principal cities in colonial India were populated by a large middle class by the second half of the nineteenth century. These included the Presidency capitals of Bombay, Calcutta, and Madras as well as large provincial towns such as Delhi, Benares, Allahabad, Lahore, Patna, Bangalore, Poona, and Ahmedabad and the hill stations where most of British Indian officialdom lived and worked for more than half the year—Simla, Darjeeling, and Ootacamund. A lively urban culture developed here; the cities and towns of British India were the locations of the successes of the colonial economy and polity, of populations of British, Anglo-Indian, and educated Indians as well as migrant laborers, traders, and small and large manufacturers. Many Indians benefited from the colonial experiments in institution building; universities and colleges, medical schools, hospitals, municipal government and local politics, and a print culture all originated and took root in the colonial cities; these in turn informed the emergent public sphere in urban colonial India.

As historians have pointed out, although the making of a modern middle class in India was a fragmented and ambivalent process, a middle-class identity was nonetheless self-fashioned through a pursuit, in many forms, of modernity.^18^ This was a conflictual and often contradictory process, involving new and older ideas of social relationships in the emergent middle-class worldview, a process that can be discerned from the late nineteenth century onward.^19^ The content of this modernity was also imbued with a deep engagement with modern science as well as ancient Indian epistemology, including medicine.^20^ Therefore, embedded as their consumption became in middle-class (however ambivalently self-conscious) households, therapeutic products assumed both political and commercial overtones; it straddled both the modern and “scientific” as well as the supposedly ancient and long-validated epistemologies and commodities. The middle classes’ own engagement with modernity was imbued with a reconstruction of the idea of antiquity and ancient tradition. While the Indian middle class had emerged from the early nineteenth-century[Other P-67] colonial establishments, the drug market in turn had developed from eighteenth-century encounters between the commerce of the East India Company (EIC) and indigenous trade. The drug market and middle-class engagement with medicine (political and therapeutic) were mutually constitutive. The two had distinctive roots.

In eighteenth-century India, both Western and indigenous medicines depended on herbs and their extracts, minerals, and animal matter. The EIC surgeons depended greatly on the local markets; their medical items, commonly known as “bazaar medicines,” included hundreds of botanical and mineral products locally available from indigenous drug sellers who were also spice merchants.^21^ Throughout the eighteenth and early nineteenth centuries, bazaar medicines constituted a significant although declining proportion of the EIC’s total expenditures on therapeutics. Later, these supplemented the total quantity of drugs used by government medical institutions. The trade in therapeutic products was differentiated according to both scale and production; at one end were the “European” drug houses that catered to the British Indian community and to the aristocratic and the affluent, at the other were the drug sellers in local bazaars who sold drugs as well as locally made generic medicines. Within this wide spectrum were the importers, traders, and manufacturers of indigenous and allopathic remedies and retailers and distributors who participated in the vast subcontinental drug market. The difference between indigenous drugs and Western ones occurred mostly in the processing stage.

In the nineteenth century, the hospitals and charitable dispensaries that were supported by the colonial state predominantly used drugs procured from England. At this time, Britain imported a huge amount of bulk drugs from India; these were then processed and re-exported at much higher prices. A large proportion of drugs were bought by the Indian army in India for the use of its troops in their hospitals. The army also extensively purchased from abroad all kinds of pharmaceutical preparations and proprietary medicines. Since the expansion of the hospital system in colonial India was first undertaken and then overseen by military administration, the supply of medicines to them was undertaken by the medical department of the government.^22^ Therefore the government was the biggest purchaser of medicines at the turn of the century. The Medical Store Depots (MSDs), located at Calcutta, Bombay, Madras, Lahore, and Rangoon, also purchased drugs and chemicals in the bazaars and[Other P-68] processed them in their own factories. The government was both manufacturer as well as supplier of medicine to the government-aided hospitals and dispensaries. Although it processed some local drugs in its factories, in the late nineteenth century the MSDs preferred to patronize firms from England. Large manufacturing firms in England had their agents in Bombay or Calcutta who negotiated contracts with government on their behalf.^23^ Until decolonization (except in the years of the two wars), the bulk of the purchases by MSDs were from Britain. This was the cause of great resentment on the part of Indian manufacturers and importers.^24^ This pressure assumed political proportions when nationalist Indians joined the Indian manufacturers in pressuring the MSDs to purchase Indian (India-manufactured Western and indigenous drug) products.^25^

## The Heterogeneous Marketplace

The private market flourished outside of government-aided institutions. In the early twentieth century, urban areas in British India became the sites of competition for the distribution and sale of numerous medicines imported from Britain, Germany, the United States, and even Japan. This also facilitated the entry and presence of several pharmaceutical multinational companies (MNCs), and the Indian market became a lucrative area for foreign companies. Some of the companies involved in exporting pharmaceutical products to India included Wellcome Burroughs (UK), Burgoyne and Burbidges (UK), Parke, Davis and Company, (United States), Merck (Germany), and Bayer (Germany). The consumer market in pharmaceutical products refers not only to drugs, but also to aerated water, hair oil, creams and ointments, toothpaste, aphrodisiacs, and innumerable tonics. The drugs included patented medicines as well as generic cholera pills, “fever pills,” “stomach pills,” and aphrodisiacs. The identification of diabetes as a widespread condition among middle-class Hindu men also raised the specter of “performative degeneration”[Other P-69] of the Hindus vis-à-vis the supposedly stronger and more virile Muslims (as well as the British), which in turn enlarged the market for “tonics” that promoted, expansively, vitality and vigor.^26^ The development and dissemination of nutrition research in the interwar years subsequently encouraged the sale of vitamin products.^27^

**Figure 1:**
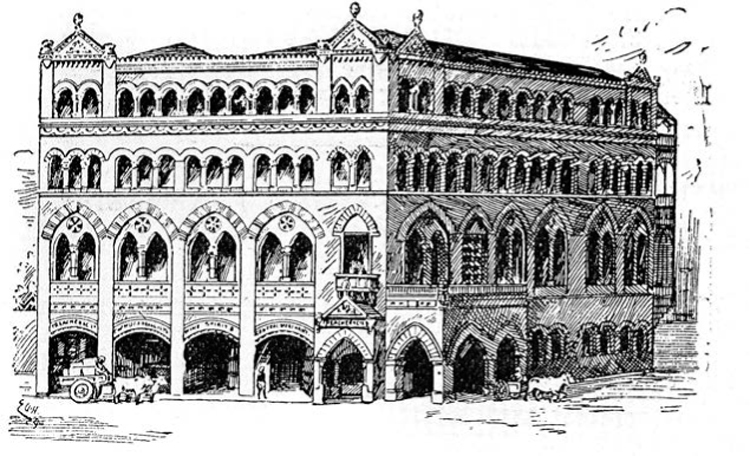
The Treacher and Company Building, Bombay, 1894. Wellcome Library, London.

In the late nineteenth century, the bulk of the drugs were sold by British or European importers. They were based in the colonial metropolises of Calcutta, Bombay, and Madras and catered mostly to the European (white) clientele both in the cities and in the hill stations, district headquarters, industrial enclaves, and commercial hubs where the British population of India resided. The largest of the agencies were Treacher and Company (Bombay and Poona, Figure 1), Kemp and Company (Bombay), Messrs Phillip and Company (Bombay), Frank, Ross and Company (Calcutta), Bathgate and Company (Calcutta), Martin and Harris (Calcutta), Stanistreet, Smith and Company (Calcutta), Symes and Company (Simla and Ambala), and W. E. Smith and Sons (Madras).

[Other P-70]

These firms sold at retail; but this was only part of their widespread business concerns. They also procured and sold wholesale drugs for large British-owned companies in India, including several rail and tea companies and collieries that dispensed basic drugs such as compounds of cinchona on a large scale to their workers, or to the native states that built therapeutic institutions for the public, such as in Baroda, Travancore, and Hyderabad.^28^ Pharmaceutical companies in Britain and Germany also appointed their own agents who negotiated contracts on their behalf with the large wholesale firms, with one agent sometimes working for two or even three firms simultaneously.^29^ So there was a complex hierarchy of manufacturers’ agents, wholesalers, retailers, and “bazaar pharmacists” who competed with each other; yet it was also a segmented market; the prosperous British residents and aristocratic Indians patronized the large, European-styled retailer–manufacturers while others resorted to bazaar traders or itinerant drug sellers of all descriptions.

British residents in India patronized “European” (British-owned) establishments to supply them with many necessities. Imports included umbrellas, cutlery, photographic equipment, perfumes, cosmetics, sewing machines and gramophones, preserved fruits, medicated wines, brandies and spirits, tobacco, canned meats, cocoa, chocolate, and, invariably, bottles of patent medicines, powders, and medicine chests.^30^ The expansion of the bureaucracy and military establishments of the Raj in the post-1858 period fostered a larger community of British consumers of European medical and allied goods. Prospective civil servants, visitors, and missionaries who might live away from urban centers were advised to purchase a “medicine chest” from a manufacturer in Britain before embarking on the journey.^31^ By 1887, distributors in Bombay and Calcutta were sending their representatives to the hill stations and cantonment towns in northern[Other P-71] and southern India, opening new branches as well as contacting medical practitioners directly. The British trade journal *Chemist & Druggist* urged ambitious pharmacists in Britain to look for opportunities in India.^32^ These pharmacies provided for Europeans as well as aristocratic Indians; most of their products were too expensive for Indian pockets, even those of the middle and professional classes.

**Figure 2:**
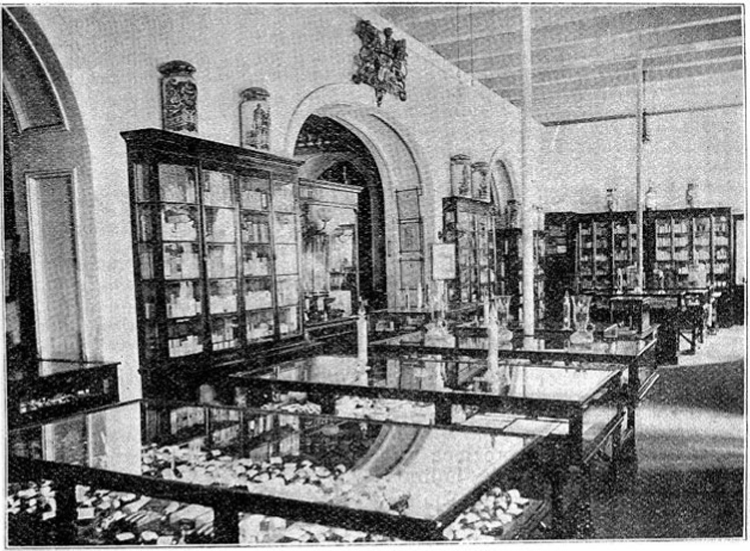
The hall of Stanistreet, Smith and Company’s pharmacy, Calcutta, 1902. Wellcome Library, London.

Although BP was the standard-bearer for drug sales in India, there was no legislation to enforce its preeminent position. In the absence of either a food and drugs law or a self-governing body of pharmacists, the quality of the goods varied; therefore, each prominent retail store depended on its own name brand for marketing. In many cantonment towns and hill stations, these stores were part of the commercial landscape. The Bombay-based Treacher and Company had achieved a virtual monopoly over European pharmaceutical products in Poona, a cantonment town near Bombay. A guidebook for visitors to Poona pointed out that the street where their outlet was located was known informally as Treacher Road.^33^ The manufacturer–importers Bathgate and Company and Stanistreet, Smith and Company (Figure 2) both featured as predominant aspects of the cityscape of Calcutta in a popular Bengali satire on British rule and its impact on India.^34^

The British Indian traders in Bombay, Madras, Rangoon, and Calcutta engaged directly with suppliers from Britain as well as their agents. One prominent agent, Mr. Charles W. White, for instance, represented Burgoyne, Burbidges and Company, A. F. Pears, as well as Wellcome Burroughs and Company in India. While he was a larger-than-life figure, well known to all British and several Indian wholesalers, he was only one of several.^35^ These distributor–manufacturers used English dailies and periodicals as well as printed catalogues and circulars posted by mail order to reach their consumers. They marketed through advertisements in newspapers and periodicals in English, evangelical periodicals, regional-language newspapers, and medical journals.^36^ In the early twentieth century, there was a boom in medical publishing in India on the back of the medical manufacturers, retailers, and agents who advertised heavily there. The[Other P-72] distributors also directly canvassed medical professionals, both official and unofficial.

The need for advertising and canvassing seems evident because of competition from Indian distributors and importers. Nonetheless, distributors of “British-made” imported pharmaceutical products enjoyed unrivalled status and an almost mystical reputation for “quality,” as did any other consumer product that was imported, especially from the metropolis.^37^ The privilege of displaying well-known British names was treasured by distributors in India. British companies provided their own labels with space for the distributors to place their own agencies’ names and addresses, highlighting both the manufacturer and the wholesaler. In spite of their reputation, the BP standard was not necessarily maintained by all British distributors because the market remained unregulated.^38^ At the turn of the century, India (along with Southern Africa and Australia) was one[Other P-73] of the developing markets for the drug trade in the global expansion of the industry, and it is not surprising, therefore, that trading within India was an emerging focus of drug exporters from Britain, America, and the Continent. There are no reliable records for the total amount of manufacture and trading in drugs and pharmaceuticals in British India at this time. But apart from various anecdotal sources, a perusal of the number of companies that paid custom duties for the import of “spirituous material,” largely for use in tinctures and drugs, provides us with the names of dozens of exporters from England, France, Germany, and America.^39^

In addition to the luxurious displays in the Europeanized commercial streets of British India, there also lurked a large bazaar market, catered to by Indian distributors and wholesalers. The term “bazaar” typically denotes an Oriental marketplace, an essentialized, chaotic, and exotic space. Historians of the marketplace in India have pointed out that the bazaar was far from a quintessentially exotic marketplace and therefore subordinate to the European capitalist market.^40^ As Pratik Chakrabarti has argued, in the eighteenth century the bazaar represented the constructed space where European merchants met with Indian interlocutors to buy local therapeutic products, often in unprocessed form, that were then traded overseas.^41^ It was also where European surgeons found “bazaar medicine” for use in their own armies and hospitals. Late Victorian pharmacists in Britain referred to all Indian traders and merchants as “bazaar traders”; it was a racialized term, imbued with the threat that Indian traders and wholesalers posed to the prominent British Indian firms. Indian importers and wholesalers often preferred to patronize German, Japanese, and American manufacturers and undercut the prices of the more prominent and visible British manufacturers and their agents and distributors in India. Indeed, as an editorial in the British trade journal *Chemist & Druggist* pointed out at the turn of the century,

The drug-trade is changing … the strictly English pharmacies do not multiply, as they are needed solely by the white people and richer natives. The bulk of the people get their medicines from the bazaar druggists, who are becoming[Other P-74] more numerous. They are keen buyers, because they are the keenest sellers in the world. … German firms are now keen competitors, … and do direct business with the bazaars.^42^

The bazaar market, in fact, had thrived in the late nineteenth century and encompassed not only botanical and mineral products, but also a great number of processed therapeutic products that included “tinctures, pills, and homeopathic medicines … and locally-made instruments.”^43^ There was a continuum, therefore, between the large global drug sellers and the so-called bazaar traders who were also a segmented lot.

The bazaar traders catered to the large numbers of middle- and lower-class urban Indians. They encompassed the occupational descendant of the spice merchant who provided bazaar medicine to the large-scale importers and distributors of European pharmaceutical products. Despite competition and racialized interactions, the bazaar traders negotiated and partnered with the more prestigious British Indian importers. The agent and proprietors of B. W. and Company, for instance, were enraged when they discovered that British importers in Madras, W. E. Smith and Sons, were passing on a part of their “unique” discount for certain branded products to the Indian distributors, H. S. Abdul Gunny and Company, of Calcutta. Their agent sent stern warnings to cease the practice; but in a diverse market, even the British Indian distributors moved beyond their European niches to participate in the bazaar trade in pharmaceutical goods.^44^

The Indian distributors proved more flexible than the large British pharmacies and resorted to importing from Germany, the United States, and Japan at cheaper rates for similar products. As a visiting American pharmacist commented in the late nineteenth century, “The patent medicine trade is large, but it is much hampered by the natives, who sell at prices that Europeans cannot touch.”^45^ In 1892, the *Indian Medical Record* estimated that in Calcutta alone there were around 756 druggists businesses and divided them into three grades: from the electrified, beautifully presented showrooms to the crowded wholesalers in the native bazaar.^46^ Similarly, Bombay had a large number of Indian druggists-cum-manufacturers. The British Indian pharmacists generally alleged that[Other P-75] bazaar pharmacists bought adulterated and inferior medical products, “cheap bazaar catch-lines from Great Britain.”^47^ Indian traders in fact often favored German products because of the attention they afforded to the market. As an Indian trader pointed out, the British trader needed to “adapt himself more to the requirements, tastes and prejudices of the millions in India,” just like the German exporters.^48^ These included looking at cultural demands in the market, from narrowing the size of marrow spoons to suit the size of Indian sheep to selling whole canned fruit because of the Hindu taboo regarding eating fruit cut by people of a different caste.

The flourishing market lent impetus to the increasingly large proportion of the non-British share of the Indian medical market in the twentieth century. Indian drug sellers began to manufacture their own products, including patent and proprietary medicines, especially the ubiquitous fever, cholera, and dysentery pills. The devastating plague epidemic of 1894–96 in Bombay resulted in drastic government public health measures and led to several indigenous “cures” that were both sold in the open market as well as peddled to government hospitals.^49^

**Figure 3:**
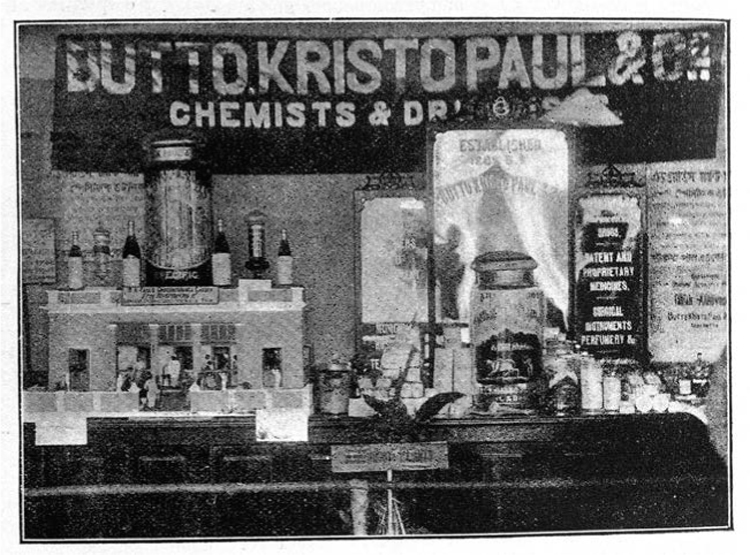
Front of Butto Kristo Paul’s stall, Calcutta, 1902. Wellcome Library, London.

One of the largest firms in India, B. K. Paul of Calcutta, was both importer and producer of medicines; it began as a small family firm and by 1905 employed around three hundred assistants in retail outlets in Calcutta alone (Figure 3). For a time before World War I B. K. Paul even enjoyed “the distinguished and (to a Bengali) rare honor of Viceregal patronage” of the governor of Bengal.^50^ The firm manufactured its own patent medicines, homeopathic medicines, surgical instruments and imported and distributed pharmaceutical products from Europe and North America.

B. K. Paul’s main retail establishment in Calcutta rivaled in display any other British Indian pharmacy. After the expansion of the company during World War I, it employed over fifteen hundred workers.^51^ Immediately after the war, the grandson of the founder, H. N. Paul, led the first short-lived professional body of pharmacists, the Calcutta Chemists and Druggists Association, which lobbied for favorable exchange rates from British banks in Calcutta.^52^ B. K. Paul featured in medical trade directories[Other P-76] published in Britain, along with Wellcome Burroughs, Boots Pure Drugs Company, Parke, Davis and Company, and the British Indian companies of Calcutta and Bombay.^53^

B. K. Paul’s success story, unique for a Bengali-owned company, has been interpreted by Mukharji as a talismanic story of Bengali entrepreneurial success that was acceptable to contemporary middle-class Bengalis as a nationalist and entirely philanthropic success. In the process, the firm and its owner, Butto Kristo, have been understood as representing a crystallization of a new kind of drugs trader, marginalizing the older, subaltern herb collectors and gatherers who have remained nameless in Bengali nationalist iconography. Mukharji’s claims are tenuous on two grounds. First, the loyalty of the Bengali middle class toward indigenous drugs was ambivalent. They were often biased in favor of foreign-produced drugs.^54^ Nationalist loyalty toward pharmaceuticals was divided, where[Other P-77] European manufactured products were highly regarded. Second, B. K. Paul continued an older tradition of family firms that dealt in wholesale markets—except that the challenges were new. The dominating presence of Anglo-Indian manufacturers and distributors lent a racial edge to the competition. Butto Kristo was not an educated pharmacist in the Western medical tradition. His success was derived from the family’s nineteenth-century presence in the bazaars.^55^

Other large Indian bazaar traders flourished as well, especially in Bombay, where indigenous capital had a freer rein than it did in eastern India.^56^ N. Powell and Company was established around 1889 by an Indian, A. L. Nair. His company traded in imported pharmaceuticals and surgical instruments and manufactured surgical instruments. In 1909, he was the only non-European importer and distributer who exhibited his products at the exhibition of the Bombay Medical Union; by this time his company was the Indian agent of several exporters too.^57^ N. Powell and Company led an informal association of Bombay chemists and druggists, especially lobbying on behalf of the Indian merchants.^58^ After World War I, the proprietor toured Britain and the Continent in order to canvass for agencies from foreign exporters.^59^ In the 1930s, the company was commended as the producers of the best-quality surgical instruments, asthma tablets, cod liver oil, chlorodyne, cough medicine, kidney pills, liver pills, lung tonic, health salt, quinine tablets, and tonics, all of which could, the nationalist Bombay Medical Union declared, replace foreign imports.^60^ Rather than bearers of cultural identities, both B. K. Paul and N. Powell need to be seen as components of a new structure of drug markets in the early twentieth century.

Meanwhile, rising nationalist aspirations from the late nineteenth century highlighted the economic exploitation of the colonial state.[Other P-78] Nationalists decried the decline of India’s manufacture as the cost of industrialization in England and advocated economic nationalism. With large-scale protests at the partition of Bengal in 1905, the swadeshi movement led a boycott of foreign goods and supported indigenous manufacturing.^61^ Throughout the nationalist struggle, swadeshi remained a great force in Indian politics. Between 1905 and 1907, several Indian chemical and pharmaceutical companies emerged; these were distinct from the druggists-cum-manufacturers. They were established by chemists who were trained in scientific techniques and interested in setting up laboratories that would manufacture pharmaceuticals to compete directly with imported pharmaceuticals. Two prominent scientists—P. C. Ray, a professor of chemistry who started Bengal Chemicals and Pharmaceutical Works (BCPW) in Calcutta, and T. K. Gajjar, also a chemistry teacher—partnered with B. D. Amin, a trader, and established the Alembic Chemical Works (Alembic) in Bombay in 1902. In 1907, Alembic moved its factory to Baroda, a princely state in western India. The generous patronage of the modernist ruler of Baroda, Maharaja Sir Sayajirao III, enabled them to set up a large factory, and their specialty was the production of alcoholic tinctures, fruit essences, and perfumery.^62^ BCPW, celebrated in independent India for its pioneering pharmaceutical production, competed directly with the MNCs as well as European distributor–pharmacists. In fact, emergent Indian pharmaceutical companies competed for the same market and manufactured both Western and Ayurvedic medicines. Some of them did not retail their products but instead concentrated on distribution of their goods wholesale to all of India. They also took the opportunity to display their products at industrial exhibitions in India, especially once the swadeshi movement for economic nationalism gained in momentum. As Lisa Trivedi has argued, the exhibitions served to delineate the geography (and the culture) of the aspiring nation.^63^ These exhibitions defined the nation as much as the imperial exhibitions of the Victorian era had defined the reach and power of the British Empire. The Indian National Congress organized the first Indian Industrial and Agricultural Exhibition in 1901 in Calcutta, and in succeeding years to Ahmedabad, Bombay, Madras, and Benares, all intensely urban manufacturing or trading centers. By the time it came around again to Calcutta in 1906–7, it[Other P-79] attracted manufacturers from all over India and included about a thousand exhibitors.^64^ The manufacturers in the soaps, chemicals, and perfumery and confectionary sections all provided Indian alternatives to imported products, with the added attraction of reduced prices for middle-class consumers.^65^ The new pharmaceutical companies like BCPW and Alembic as well as the older importer–manufacturers like B. K. Paul won awards for the best quality products in therapeutic goods.^66^ Whereas the firms established by modern chemist–nationalists like P.C. Ray and T. K. Gajjar were not classed as producing “bazaar medicine,” those of B. K. Paul and N. Powell, who were distributors as well as manufacturers, definitely fell into that category. The nationalist activism of Ray and Gajjar has served to highlight their importance in Indian industrialism in nationalist discourse. Historians have also argued that they both were pioneers (particularly Ray, a nationalist) of the pharmaceutical industry in British India.^67^ They have neglected to indicate that their entrepreneurship was part of a broader trajectory that included manufacturers becoming drug importers and dealers. There was a continuum, therefore, from the so-called bazaar dealers to the scientifically trained manufacturers that involved importers, distributers, and producers. The critical element of this market was not the heterogeneity of its sellers, although that was relevant, but that a single drug could be imported or processed, distributed, and consumed in many different forms, potencies, and prices, very seldom in conformity with BP. The most obvious example of this is the trade in quinine and its alkaloids—these were sold in several potencies and differing prices across the country.^68^ There was an inherent lack of standardization.[Other P-80]

## Adulteration in a Heterogeneous Market

One reason for widespread adulteration was that government regulation was light. There was no food and drugs law in colonial India; laws regulating the sale of poisons and narcotics like cocaine evolved piecemeal. In Bengal all pharmacists selling British pharmaceuticals containing any form of poison had to be registered, but this law hardly precluded hundreds of sellers from trading in the informal market. In Bombay, a similar act could be enforced only on the pharmacies in the metropolis, and then only the larger ones.^69^ For medico-juridical purposes, the government appointed three chemical examiners, at Bombay, Calcutta, and Madras, whose duty, among many, was to examine foods and other substances sold in the market and those seized by authorities, usually in cases of grave illness or the death of a consumer. The government’s all-India Poison Act was passed in 1904 after a decade of deliberation; its implementation was fragmentary at best.^70^

Among the elite medical officials of the Indian Medical Service, there were discussions on limited regulation of the drug market. These continued until the interwar years, when public pressure fueled by Indian newspapers and lobbying by the most prominent British and Indian manufacturers succeeded in the appointment of a drug standardization committee by the government in 1930. Effectively, there was no control over the drug market in late colonial India.

In official discourse, the preexistence of a large, informal market in variable drugs were supposed to have prevented any legislation to control it. In 1894, at the very first the Indian Medical Congress at Calcutta, two IMS officials, one a Bengali physician, read a paper together on the sale and “ease of distribution” of poisons in India. They pointed out that the chemical examiners at Calcutta and Madras particularly had found hundreds of deaths by poisoning and urged the need for legal control. But the elite IMS officials claimed that it was “Utopian” for India to have a poison schedule similar to Britain’s.^71^ Even after the Indian Poisons Act was enacted, the *Bombay Gazette* highlighted that only druggists selling BP products were covered in terms of the law, whereas Indian medicines were exempt, and moreover, “Bunneahs and small dealers sell any number of drugs.”^72^ Whereas the Poisons Act referred only to poisons[Other P-81] and narcotics, the quality of drugs or other products was not regulated. These varied enormously and were a rich source of contention between competing European importers as well as among Indian dealers. The bulk of the blame for the preponderance of substandard products fell on the bazaar traders, from the large wholesale importer to the modest pavement drug seller.

The adulterated market was vast; it included imitations of well-known imported branded products like soaps, powders, and patent medicines as well as bulk products like olive and almond oils. In 1889, Charles W. White, the British agent for B. W. and Company, A. F. Pears and Company, and Burgoyne, Burbidges and Company of England, claimed on one visit to India that German, French, and American competition, with the full complicity of the Indian wholesalers, was marginalizing the British manufacturers with brazen adulterated oils and powders: “calomel containing 50% of chalk, santonin half boracic acid, sent here from France and Germany carriage paid and sold in the currency of the country, I fail to see how honest British competition can stand.”^73^ White successfully challenged the sale by dealers in Bombay of the popular Pears glycerin soap, allegedly supplied from Germany.^74^ In the next year, Bertie Smith, a British wholesale importer of drugs based in Bombay, identified “German competition” as his problem, and accused Continental firms of monopolizing trade in certain drugs by exporting cheap, adulterated material to “native” drug importers.^75^ An anonymous Indian correspondent of the British trade journal *Chemist & Druggist* claimed that an “American-made bazaar counterfeit” of an English brand of patent drug made from sarsaparilla flourished in the Indian market.^76^

In 1905, an impassioned appeal by the Parsi physician Dosabhai Rastamji Bardi, who taught at the Grant Medical College in Bombay, demonstrated the extent of the adulteration in food and drugs. Citing records of the Chemical Examiners of Bombay from 1872 to 1902, Bardi claimed that there was consistent adulteration of imports to the Indian market:

It is necessary to remember that the retail druggist hardly adulterates them, but as people want cheap drugs, he buys adulterated articles … no wonder that medical men are disappointed in their treatment. Bombay, and for the matter … the whole of India, depends on European and American markets for their supply of drugs, at any rate of all important pharmaceutical and chemical preparations.^77^[Other P-82]

Colonial historians have not engaged with the problem of adulteration in the Indian drug market. Contemporary analysis of the Indian drug market was one of adulteration and the lack of drug control; in both official and medical discourse, adulteration was linked largely to the bazaar market, Indian manufacturers of both Western and Indian medicine, and unscrupulous importers who dealt with products from the Continent. The official and medical rhetoric suggested that the respectable end of the market was dominated by British Indian manufacturers and importers. The professionalization of pharmacy is also the predominant theme of analyzing the history of the drug industry in developed Western nations.^78^ Although adulteration or the presence of spurious or diluted medicines was an important reality of the Indian market, the lack of professional education and self-regulation and corporatization among pharmacists provides only a partial analysis of adulteration. What is omitted from the analysis of adulteration by contemporary commentators (and indeed the rare historian such as Barton has addressed it with reference to quinine) is the need for drugs of varying prices to suit the different sections of consumers within the Indian market. This led to drugs of different strengths and potencies, often in disguised form, because these differing potencies were not recognized by BP.

The case was even more complicated by the widespread use of indigenous drugs, which could not be standardized according to BP because Western medical professionals suggested that their active principles should first be analyzed, a gigantic and impossible task. Adulteration therefore covered a spectrum of deficiencies among the drugs sold in the market; some were willfully and totally fake medicines, others were simply drugs of a lower potency and price.

Examples of adulteration in imports found by the chemical examiner of Bombay included the presence of hydrochlorate of cinchonin, a much cheaper product, in a sample of quinine sulphate, potassium nitrate containing hydrochloric acid, worm tablets with no santonin, and a sample of tartar emetic not conforming to BP tests.^79^ Importers provided substandard products not only to retail druggists, but also to the government’s MSDs. Adulteration and substandard goods was pervasive; the diversity and quantity of the therapeutic products on sale in India appeared to defy[Other P-83] any serious attempt at regulation. BP was the yardstick of quality, and a “colonial addendum” to it in 1898 legitimized the “substitution” of several drugs for those that were easily available and commonly used in India by Western practitioners.^80^ This addendum was the consequence of a long campaign in India by several eminent medical personnel, both British and Indian.^81^ British pharmacists alleged here that the addendum was of only academic value: “*In India there is no Pharmacy Act; a Pharmacopeia is looked upon more as a guide*.”^82^

The fifty-four drugs added to BP in acknowledgment of indigenous drugs that could be prescribed by Western practitioners in India made little difference to the production or sale of indigenous drugs *instead* of imported ones; and indeed, the major thrust of import substitution and production of indigenous drugs began only when World War I severely circumscribed the import trade in the Indian drug market.

In this market, therefore, manufacturers and distributers relied heavily on branding and advertising. All major drug companies, both British-and Indian-owned, warned against imitations and substitutions cleverly produced to fool customers into buying substandard goods. What then defined purity to the customer? The pricing provided a guidance of sorts: expensive products were considered more efficacious. Even this standard was subverted on a regular basis with the production of imitation drugs sold in reused packaging from reputable manufacturers, who responded by printing warnings against imitation products in advertisements and circulars.^83^ The Indian public, meanwhile, believed that the British firms sold their outdated stock at cut-rate prices to bazaar merchants.^84^

Drug traders provided cheap products to consumers everywhere, and there was a wide spectrum between “pure” and “impure” drugs that embraced several degrees of authenticity. The only drug that the government attempted to distribute widely in India was quinine and its alkaloids, at first distributed free, and then sold at a nominal price. Through inexpensive packets sold at post offices and later through a wide-ranging network of distributors, provincial governments sought to limit the devastation[Other P-84] caused by malaria. As Patricia Barton has demonstrated, the high levels of adulteration (up to 80 percent) of the quinine tablets sold by government agents and other distributors subverted the policy.^85^

Meanwhile, Indian druggists faced most of the blame for adulteration and substandard products in the market. These might include imported patent medicines as well as drugs commonly used in both indigenous and Western systems of medicine, including belladonna, aconite, senna, and asafetida. In 1910, the *British and Colonial Druggist* suggested that while “the native vendor is keen after a bargain, … when it is said that he can purchase Easton’s syrup in four oz. bottles, each packed in a carton at 36s. gross, it appears that the limit of cheapness has been reached.”^86^

The hugely popular Moore’s medical manual, a handbook of medicine and diet for British residents in India that went through several editions, cautioned in 1916 that “the adulteration and sophistication of the specimens found in the Indian market, and the ariability … of the drugs themselves … renders it quite impossible to use them with safety.”^87^ From the standpoint of both the “legitimate” trades and official medicine, therefore, the problem was twofold: dilution and imitation of standard drugs and the uncontrollable and uncertain quality of the fresh drugs on the market that problematized the indigenous drug market.

Before World War I, public discourse in India recognized that some kind of legislation was needed, and in the Upper Provinces one Indian councilor referred a request for legislation to the Select Committee in 1911.^88^ That same year the *Statesman*, a Calcutta daily, began a campaign against spurious drugs, many of which included diluted or impure drugs from Britain, the United States, Germany, and Japan. The debate began with a letter from Norman Hirst, a pharmacist who alleged that “they send out to India cheap varieties of quinine sulphates; compound extracts of sarsaparilla in some instances consisting chiefly of glucose and many other medicinal preparations … are almost entirely deficient in active principle and are practically inert.”^89^ Hirst demonstrated the complicity of both the consumers and the exporters and alleged that the British Indian companies needed drugs on a large scale for their laboring populations, such as “tea gardens, railway companies, collieries, … send round ‘tender forms’ or quotations for their medicines, and they usually accept the[Other P-85] lowest tender.”^90^ Therefore adulteration in the Indian market needs to be understood as a consequence of not simply the traders’ rapacity or lack of government control, but also the differential needs and priorities of the consumers of therapeutic products.

In the age of nationalism, Indian medical professionals often tended to view adulteration at two levels: the failure of self-regulation by the merchants and industrialists and the lack of a professionalization by pharmacists. The *Indian Medical Record* argued for cooperative associations and suggested the establishment of an apprentice system to train prospective pharmacists.^91^ Both were distant dreams. The popular movement for a drug control policy continued in the Indian press, both national and regional. In the interwar years it proved a nationalist issue. The Indian press, in a time of intense competition between Indian and British capital in manufacturing and trade, inverted the charge of adulteration and pointed out that British manufacturers and British importers in India colluded to release substandard pharmaceutical products in the Indian market. In 1925, an article in the daily the *Bengalee*, reproduced by the *Indian Medical Record*, alleged that all reputed pharmaceutical companies in Calcutta, both British-owned and Indian, made a regular practice of misleading customers by labeling their products as being of BP strength, including BCPW, Stanistreet, Smith and Company, D. Waldie and Company, and B. K. Paul (distributors of imported products as well as manufacturers). It claimed that the chemical examiner of Calcutta had reported that “local firms in competition with one another and with the importing firms try to reduce the manufacturing cost by using less medicament and alcohol, and that the importing firms in their turn have begun a similar practice.”^92^ After a strong protest from the Stanistreet, Smith and Company, the *Indian Medical Record* retracted this piece, but the all-pervasive collusion of manufacturers, producers, and distributors in various stages of adulterating drugs was apparent and raised continual pleas for a food and drugs act for India. This was particularly aggravated in the immediate postwar era, when British, German, and American companies dumped excess World War I stocks on the Indian market on a large scale. In the interwar years, therefore, while most problems of adulteration remained familiar, they acquired a new urgency in public discourse.

Why was adulteration so pervasive and impossible to contain? One may argue that the market itself settled for differing degrees of potency, sold at[Other P-86] correspondingly different rates to suit the pockets of consumers. But the medical market (like any other in colonial India) in effect was not laissez-faire. The MSDs bought a large quantity of medical products (from the United Kingdom) and manufactured several drugs in their factories. The Indian army and on a lesser priority the government hospitals, therefore, were provided with a quality of drugs that usually conformed to the BP standard. Until World War I, therefore, the government of India showed little interest in controlling the private market in drugs and remained indifferent to public opinion or nationalist pressure for a drug control law.

World War I changed government priorities. During the war the Indian army was deployed to several places, such as Mesopotamia, where imports from Europe proved difficult and those from Germany ceased altogether. The MSDs’ own produce proved inadequate; they relied on manufacturers based in India to provide several standard drugs and surgical products. Several Indian manufacturers, particularly the large producer–retailers, made a fortune in supplying government contracts.^93^ The MSDs ordered from the most prominent British Indian manufacturers in Calcutta and Bombay. Nonetheless, during the war, the Indian army could no longer remain insulated from the variations in potency of medicine and surgical products that pervaded the private market. There was one prosecution; the Royal Army Medical Corps put Phillips and Company of Bombay on a perpetual blacklist after investigations revealed that it had supplied highly adulterated and substandard medicine and surgical dressings during the war.^94^

It was not only that the Indian army had been endangered on the field. Most government hospitals and lesser charitable institutions and private hospitals faced an acute shortage of medicines in the latter years of the war. The clamor for an independence from imports, however qualified, resonated even among British medical professionals of the IMS. Here the Indian nationalists were joined by others motivated by a huge scarcity of medicines. The urgency in public discourse reflected that within the medical profession itself. Their concern encompassed three related themes: the prevention of adulteration of drugs both generic and proprietary; professional training for chemists; and an impetus, particularly by Indian medical professionals trained in Western medicine as much as by Ayurvedas and Hakims, to classify, process, and use Indian substitutes of[Other P-87] Western imported products as much as possible. With the emphasis on import substitution and deeper exploration of indigenous drug plants (shorn of their “impurities”) by medical men and entrepreneurs alike, the complexity of the market intensified at this time. The huge range of indigenous drugs and the local manufacture of drugs came to official and medical notice; but public discourse and medical disquiet on the lack of regulation in the market grew in the immediate postwar period. Indigenous drugs were therefore both sought-after and the objects of suspicion. Although the demands for import substitution of drugs from the indigenous pharmacopeia continued to inform government policy, once the desperate urgency of scarcity was over with the war, there was little conviction in any government initiative to introduce import substitution through encouraging supplies from Indian manufacturers to the MSDs. Several provincial governments (after the provincialization of health in 1919) did attempt desultory “experiments” with growing other plants such as linseed, soya, mustard, ipecacuanha, and cannabis in Sind, Punjab, Assam, and the Central Provinces. The results were fragmentary at best.^95^

After World War I, British, American, and even German imports resumed and the short-lived government emphasis on import substitution dwindled. Large-scale nationalist pressure and a push toward import substitution led to some concessions for Indian industry after the war. These did not extend to the pharmaceutical industry, however. The MSDs continued to control all supplies to government hospitals and imported drugs manufactured abroad. Government hospital administrations often resented the loss of independence and the red tape involved in their sourcing exclusively from the MSDs. In 1935, the surgeon-general of the government of Bombay surrendered to pressure from government hospitals to purchase their own medicines through inviting tenders. Several British, British Indian, and bazaar companies supplied to the hospitals, but this system was discontinued because the surgeon-general ruled that the method was liable to fraud. Therefore the MSDs’ control over the supply of government hospitals remained intact. This left the private markets vulnerable to spurious drugs, proprietary medicines that survived on aggressive and false advertisement, and adulteration at different levels of manufacture and distribution.

As we have seen, the problem of adulteration had aroused public opinion in the early twentieth century. It became prominent in nationalist[Other P-88] discourse during and after the war, when Indian companies alleged that foreign companies were dumping substandard goods in the Indian market. Nationalists intervened to demand legislation.^96^ Medical professionals both Indian and British Indian showed an interest in import substitution as well as the compilation of an Indian Pharmacopeia.

In the interwar period, when the League of Nations initiated international cooperation on both the control of narcotic drugs and the standardization of sera and vaccines, the government capitulated to the general clamor at home as well as to the new international initiatives and instituted the Drugs Enquiry Committee (DEC) in 1930. It was chaired by R. N. Chopra, who had extensively researched the properties of indigenous drugs at the Calcutta School of Tropical Medicine. The DEC, which consulted the nascent industry, retailers, and medical professionals, made several recommendations to regulate the import, sale, and manufacture of pharmaceutical products in the country and to streamline the training of pharmacists in technical institutes in its report in 1931. The government acted on the bulk of these recommendations only when it passed the Drug Act in 1944.

## Conclusion

Why was the DEC’s report shelved for fifteen years? Barton has argued that the transfer of the responsibility for health to provincial governments (which, after devolution of power in 1919, was ruled by nationalist governments) made an all-India policy impossible for the colonial government in Delhi.^97^ Control over the Indian drug industry was a more complicated affair than the lack of central government initiatives or a lack of standardization of drugs internationally, although these too played a part. The culture of the dispensation of drugs in India necessarily involved manufacturer–retailers, bazaar traders, and a multitude of medical professionals practicing a range of therapies, allopathic, homeopathic, or Ayurveda or Unani, who also sold their own potions and pills to their patients. While the Western therapeutic products could be theoretically held to the BP standards, Western medical practitioners, British and Indian alike, insisted that indigenous drugs could not be standardized because their active principles had not been isolated. Many nationalist Western-educated medical professionals campaigned for an Indian Pharmacopeia, which would include the hundreds of drugs, they insisted, that were available in India[Other P-89] and were being exported for processing abroad. Like drug control legislation, an Indian Pharmacopeia also therefore became a nationalist political demand after World War I. At this time the huge and unregulated drug market in India continued to flourish and the debates on standardization and adulteration were fused; medical practitioners and official authorities claimed that until the active principles of indigenous drugs were all identified and “scientifically” tested, it was impossible to bring them (or their practitioners) within a regulated market. The demands for import substitution of drugs from the indigenous pharmacopeia continued to inform government policy, albeit in altogether a too lackadaisical manner to invoke any conviction in the policy among consumers. An indigenous drugs manufacture committee was formed in 1920, and it diligently reported on the progress on manufacture to facilitate import substitution until 1923.^98^ At this time the export trade to India picked up again, and import substitution reverted to being a nationalist aspiration rather than government policy, until the next world war.

The imported medicines suffered from no such lack of scientific authority as the Indian substitutes. Instead, in their case, patent medicines remained the crux of public discourse and medical authorities’ consternation.^99^ The DEC had condemned the huge trade in patent medicines as surviving on aggressive and usually false advertising and on the gullibility of medical practitioners as well as consumers, and most of these related to imported products. When adulteration became a fiercely contested issue between the British Indian distributors and the Indian manufacturers, it affected the import trade on two counts. The first was a general, nationalist push for the consumption of swadeshi goods. Second, the discourse of the distinctiveness of the tropics as a unique disease environment (that defined tropical medicine) permeated in the popular and even medical imagination. This led to the idea that only therapeutics manufactured in India were suitable for Indian bodies and the Indian climate.^100^ While this trope was favored by Ayurvedas and Hakims, practitioners of Western medicine fused these cultural-climatological ideas within their medical practice as well. For instance, Dr. Bose’s Laboratory, a manufacturing pharmacy set up in Calcutta by Kartick Chandra Bose, a physician–entrepreneur, advertised its own products as being superior to imported goods,[Other P-90] because certain drugs (such as lactic acid pills) deteriorated entirely by the time they were consumed in “tropical” India.^101^ Bose also founded a medical journal, *Food and Drugs*, in which he advertised his own varied products and those of B. K. Paul. *Food and Drugs* published articles related to the beneficial “active components” of Indian fruits and vegetables commonly used and available in the bazaars.^102^ Therefore the issue of adulteration was fused with logistic issues of spoilage and climatic and racial tropes and was contested fiercely by rival manufacturing and trading firms. While the government research institutes continued to produce sera and vaccines on a large scale with a focus on their standardization, there was no such control over the quality of drugs for sale in the open market. Adulteration and its elusive counterpart purity remained contested sites in the medical market in colonial India.[Other P-91]

## Footnotes

1
BartonPatricia‘The Great Quinine Fraud’: Legality Issues in the ‘Non-Narcotic’ Drug Trade in British IndiaSoc. Hist. Alcohol Drugs2212007625

2
Davenport-HinesR. P. T.SlinnJ.Glaxo: A History to 1962CambridgeCambridge University Press1992 LiebenauJonathanMedical Science and Medical Industry: The Formation of the American Pharmaceutical IndustryBaltimoreJohns Hopkins University Press1987 QuirkeV.Collaboration in the Pharmaceutical Industry: Changing Relationships in Britain and France, 1935–1965New YorkRoutledge2008 RobsonMichaelThe French Pharmaceutical Industry, 1919–1939Pill Peddlers: Essays on the History of the Pharmaceutical IndustryHigbyGregory J.StroudElaineLiebenauJonathanMadisonAmerican Institute of the History of Pharmacy1990107121 ChurchRoyTanseyE. M.Burroughs, Wellcome & Co.: Knowledge, Trust, Profit and the Transformation of the British Pharmaceutical Industry, 1880–1940LancasterCrucible2007

3
SlinnJudyResearch and Development in the UK Pharmaceutical Industry from the Nineteenth Century to the 1960sDrugs and Narcotics in HistoryPorterRoyTeichMikulášCambridgeCambridge University Press1996168186

4
SivaramakrishnanKavitaOld Potions, New Bottles: Recasting Indigenous Medicine in Colonial Punjab, 1850–1945HyderabadOrient Longman2006 SharmaMadhuriIndigenous and Western Medicine in Colonial IndiaNew DelhiFoundation Books2012 LeslieCharlesThe Ambiguities of Medical Revivalism in Modern IndiaAsian Medical Systems: A Comparative StudyLeslieCharlesBerkeleyUniversity of California Press1976356367

5
SivaramakrishnanOld Potions, New Bottles13

6
AlaviSeemaUnani Medicine in the Nineteenth Century Public Sphere: Urdu Texts and the *Oudh Akhbar*Indian Econ. Soc. Hist. Rev422005101129

7
AttewellGuyRefiguring Unani Tibb: Plural Healing in Late Colonial IndiaNew DelhiOrient Longman2007

8
BergerRachelAyurveda and the Making of the Urban Middle Class in North India, 1900–1945Modern and Global Ayurveda: Pluralism and ParadigmsWujastykDagmarSmithFrederick M.AlbanyState University of New York Press2008101115

9 GOI Medical, “GMC: A Report from the Pharmaceutical Committee on the Proposed Indian and Colonial Addendum to the BP of 1898” (March 1899), Asia, Pacific and Africa Collections, British Library (henceforth APAC), IOR/P/5645, 373–97.

10
The Indian and Colonial Addendum to the British PharmacopeiaIndian Med. Gazette3619016263PMC516401729004069

11
MukharjiProjit BihariPharmacology, ‘Indigenous Knowledge,’ Nationalism: A Few Words from the Epitaph of Subaltern ScienceThe Social History of Health and Medicine in Colonial IndiaPatiBiswamoyHarrisonMarkLondonRoutledge2009195212

12
MukharjiProjit BihariNationalizing the Body: The Medical Market, Print and Daktari MedicineLondonAnthem Press201175110

13
SharmaIndigenous and Western Medicine120139

14
KumarDeepakUnequal Contenders, Uneven Ground: Medical Encounters in British India, 1820–1920Western Medicine as Contested KnowledgeCunninghamAndrewAndrewsBirdieManchesterManchester University Press1997172190 QaiserNeshatPolitics, Culture and Colonialism: *Unani’s* Debate with *Doctory*Health, Medicine and Empire: Perspectives on Colonial IndiaPatiBiswamoyHarrisonMarkLondonSangam Books2001317355

15
ChakrabartiPratikEmpire and Alternatives: *Swietenia febrifuga* and the Cinchona SubstitutesMed. Hist541201075942004626510.1017/s0025727300004324PMC2793143

16
AttewellGuyInterweaving Substance Trajectories: *Tiryaq*, Circulation and Therapeutic Transformation in the Nineteenth CenturyCrossing Colonial Historiographies: Histories of Colonial and Indigenous Medicines in Transnational PerspectiveDigbyAnneErnstWaltraudMukharjiProjit B.Newcastle upon TyneCambridge Scholars Press2010120

17
WhyteSusan ReynoldsVan Der GeestSjaakHardonAnitaSocial Lives of MedicinesCambridgeCambridge University Press200231979103

18
HaynesDouglas E.McGowanAbigailIntroductionTowards a History of Consumption in South AsiaHaynesDouglas E.McGowanAbigailRoyTirthankarYanagisawaHarukaNew DelhiOxford University Press2010125

19
JoshiSanjayIntroductionFractured Modernity: Making of a Middle Class in Colonial North IndiaJoshiSanjayNew DelhiOxford University Press2001122

20
ChakrabartiPratikWestern Science in Modern India: Metropolitan Methods, Colonial PracticesDelhiPermanent Black2004 VisvanathanShivA Carnival for Science: Essays on Science Technology and DevelopmentDelhiOxford University Press1997 ArnoldDavidEveryday Technology: Machines and the Making of India’s ModernityChicagoUniversity of Chicago Press2013

21
HarrisonMarkMedicine in an Age of Commerce and Empire: Britain and Its Tropical Colonies, 1660–1830OxfordOxford University Press201022

22 India Office, “Report of the Committee on the Supply of Drugs for India” (1875), APAC, IOR/L/MIL/7/15141.

23
Medical Supplies to Local and Municipal BoardsIndian Med. Rec61894148

24 For instance, see representations from Smith, Stanistreet and Company and the Union Drug Company to the Drugs Enquiry Committee in 1930, “Report of the Drugs Enquiry Committee” (Calcutta, 1931), 131.

25 Complaints from Indian manufacturers that the government stores department refused to patronize their products, not only medicine but also steel and machine tools, were continually propagated by the nationalist Indian Merchants Chamber in the interwar years. See chairman’s address, “Annual Report of the Indian Merchants Chamber, 1922–23” (Bombay, 1923), 49.

26
ArnoldDavidDiabetes in the Tropics: Race, Place and Class in India, 1880–1965Soc. Hist. Med222009245261

27
MitterK. C.IMR321912ix Notes on New PreparationsMed. Digest2193458 Glaxo Lab. Ltd.Calcutta Med. J421945ix

28 The prosperous, princely state of Baroda, for instance, regularly used both the pharmaceutical manufacturing and trading firms of Treacher and Co. and Kemp and Co. of Bombay to order the delivery of medicines, chemicals, telescopes, opera glasses, cigars, and wines and spirits. Baroda Record Office, Huzoor English, 99/127/19, 1875–79.

29
Pharmacy in IndiaChemist & Druggist1021886448 Bombay Medical Congress from the Pharmaceutical Point of ViewPharmaceutical Journal and Pharmacist3201909409 Personal ParagraphsIndian and Eastern Druggist7192025

30
ArnoldEveryday Technology4068

31
HoggFrancisPractical Remarks Chiefly Concerning the Health and Ailments of European Families in India, with Special Reference to Maternal Management and Domestic EconomyBenaresMedical Hall Press1877iii

32
Indian Pharmaceutical NewsC&D291886235 Pharmacy in IndiaC&D291886448

33
Guide to Poona and Kirkee, with Directory, for the Season 1876, etcBombayTreacher and Co1876

34
RaiDurgadasDebgoner Mortye AgamanCalcuttaGurudas Chattopadyay and Sons1929498502548

35
Pharmacy in IndiaC&D301889239

36 For instance, see *Bombay Guardian*, January 5, 1901, 1.

37
FuredyChrisBritish Tradesmen and Shopkeepers of Calcutta, 1830–1900Yorkpublished by author19769 ComptonHerbertIndian Life in Town and CountryLondonG. Newnes1904145

38 “Report of the Drugs Enquiry Committee” (n. 24), 86.

39
Alphabetical List of Drugs, Medicines and Other Preparations Free from, and Containing, Spirit, Compiled from the Results of Tests Made by the Customs Houses at Calcutta, Bombay, and MadrasMadrasSuperintendent of Government Press19031

40
RayRajat KantaAsian Capital in the Age of European Domination: The Rise of the Bazaar, 1800–1914Mod. Asian Stud29371995449554

41
ChakrabartiPratikMedical Marketplaces beyond the West: Bazaar Medicine, Trade and the English Establishment in Eighteenth Century IndiaMedicine and the Market in England and Its Colonies, c.1450–c.1850WallisPatrickJennerMarkBasingstokePalgrave Macmillan2007196215

42
Current News on Export Trade: India and CeylonC&D661905375

43
A Report of the Exhibits of Drugs, Chemicals, Instruments, etc. in the Calcutta International ExhibitionC&D261884115

44 B. W. and Co. to W. E. Smith, March 28, 1906 (henceforth WA[Wellcome Archives]), WF/E/03/21, box 114.

45
FalckJohn A.Pharmacy in IndiaC&D301887441442

46
The Chemist and Druggist Trade in CalcuttaIMR3911892350

47
Indian IncursionsBritish and Colonial Druggist3311911257 Pharmacy in IndiaC&D331888552 Indian Drugs InquiryC&D1931249

48
India and the German TradersIndian Trade Journal4161908100101

49
MukharjiNationalizing the Body179212

50
The History of a PharmacyIMR291909185

51 *C&D* 104 (1926): 757–60.

52 *C&D* 93 (1921): 70.

53
O’MearaE. J.Medical Guide for India and Book of PrescriptionsLondonButterworth1920647648

54
BanerjeaS. B.Drugs in IndiaIndian and Eastern Chemist2194039

55
GuhaMeeraGrowth of Calcutta’s Business DistrictEcon. Polit. Weekly17461113196516951698

56
BagchiA. K.Private Investment in India, 1900–1939LondonRoutledge2000157216 TomlinsonB. R.Colonial Firms and the Decline of Colonialism in Eastern India 1914–47Mod. Asian Stud1531981455486 MarkovitsC.Indian Business and Nationalist Politics 1931–31: The Indigenous Capitalist Class and the Rise of the Congress PartyCambridgeCambridge University Press198510

57
Bombay Medical Congress: From the Pharmaceutical Point of ViewPharmaceutical Journal and Pharmacist3201909409

58 *C&D* 96 (1922): 45.

59 *IED* 2 (1921): 194.

60
All India Swadeshi DirectoryAhmedabadGujarat Sahitya Mandir1931385388

61
SarkarSumitThe Swadeshi Movement in Bengal, 1903–1908RanikhetPermanent Black2011

62
AminB. D.The Rise and Growth of the Alembic Chemical Works: A HistoryBarodaAlembic Printing Press193925

63
TrivediLisa N.Visually Mapping the ‘Nation’: Swadeshi Politics in Nationalist India, 1920–1930J. Asian Stud621220031141

64
A Report of the Indian Industrial and Agricultural Exhibition, Calcutta, 1906–7CalcuttaIndustrial India Office1907v

65
A Report of the Indian Industrial and Agricultural Exhibition, Calcutta, 1906–7CalcuttaIndustrial India Office1907xxvii

66
A Report of the Indian Industrial and Agricultural Exhibition, Calcutta, 1906–7CalcuttaIndustrial India Office1907106113

67
ChakrabartiPratikScience and Swadeshi: The Establishment and Growth of the Bengal Chemical and Pharmaceutical Works 1893–1947Science and Modern India: An Institutional History, c.1784–1947GuptaUma DasDelhiPearson2010117142 TripathiD.MehtaM.Business Houses in Western India: A Study in Entrpreneurial Response, 1850–1956LondonJaya Books1990116130

68
Profits on the Manufacture of QuinineLancet991905801802 BartonPatriciaPowders, Potions and Tablets: Politics, Science and the Purity of Drugs in British India, 1890 to 1939Drugs and Empires: Essays in Modern Imperialism and Intoxication 1500–1930MillsJamesBartonPatriciaBasingstokePalgrave Macmillan2007142161

69
AllenA. C.Note on the Indian Poisons ActPharmaceutical Journal and Pharmacist8281909309 The Indian Poisons ActPharmaceutical Journal and Pharmacist10161909470

70 Allen, “Note on the Indian Poisons Act” (n. 69).

71 *Bombay Gazette*, January 4, 1895, 4.

72 Ibid.

73 *C&D* 34 (1889): 239.

74 Ibid.

75
A Talk on the Indian Drug TradeC&D37189071

76
Proprietaries in IndiaC&D5518991077

77
Bombay Medical and Physical Society Proceedings91905561

78
MalleckD. J.Professionalism and the Boundaries of Control: Pharmacists, Physicians and Dangerous Substances in Canada, 1840–1908Med. Hist48220041751981515110310.1017/s0025727300007389PMC546338 SavageDeborah AnneThe Professions in Theory and History: The Case of PharmacyBus. Econ. Hist2321994129160 KurzerFrederickGeorge S V Wills and the Westminster College of Chemistry and Pharmacy: A Chapter in Pharmaceutical Education in Great BritainMed. Hist5120074775061793255710.1017/s0025727300001770PMC2002594

79
Bombay Medical and Physical Society Proceedings9190527

80 GOI Medical, “GMC” (n. 9), 373–78.

81 See the appeal of Kanny Lal Dey in the first Indian Medical Congress in 1895, *Pharmaceutical Journal and Transactions* 25 (1894–95): 446.

82 *C&D* 62 (1903): 117.

83 For instance, in 1912 B. W. and Co. published pamphlets in Tamil, Bengali, and Gujarati advertising “tabloid” brand medicines, urging, “Beware of imitation. Take particular care to buy ‘Tabloid’ medicines which are prepared by BW and Co only.” Circular Book 18, 1912–13, WA, WF/M/GB/01/19/01, 20–23.

84
RaiDebgoner498

85 Barton, “Powders, Potions and Tablets” (n. 68).

86 *British and Colonial Druggist*, August 26, 1910, 183.

87
SprawsonC. A.Moore’s Manual of Family Medicine and Hygiene for IndiaLondonJ. A. Churchill19161

88 *C&D* 78 (1911): 38.

89
Drugs for IndiaC&D78191154

90 Ibid.

91
Co-operation as Applied to Industrial, Particularly to Pharmaceutical EducationIMR4119217980

92
Adulteration of DrugsIMR451925336

93 Export of quinine to Khartoum, Smith, Stanistreet and Co (May 1919), no. 98, 1, National Archives of India, Commerce and Industry, P/9863; Revenue/Commerce and Industry, B Proceedings (June 1915), APAC, 137.

94 “Supply of Defective Medical Stores by Messrs Phillips and Co. Bombay, 1916,” APAC, IOR/L/MIL/7/15148/8.

95
Medicinal Plants in MadrasIED619295

96 Dharamsi Morarji and Company to the Government of India, *Indian Medical and Pharmaceutical Review*, February 1924, 33.

97 Barton, “‘Great Quinine Fraud’” (n. 1).

98
Report on the Proceedings of the Drugs Manufacture CommitteeSimlaGovernment Central Press192015

99 National Archives of India (n. 95); “Medicinal Plants in Madras” (n. 95), 5.

100
Illicit ImportationIED2193029

101
On the Danger of the Use in India of So-Called Lactic Acid Bacilli Tablets Manufactured in EuropeFood and Drugs119101522

102
GhosalLal MohanAn Investigation into the Chemical Composition and Properties of the Milk (Water) of the CocoanutFood and Drugs1191033 The Study of Metabolism of Mango …Food and Drugs119108084 TurnerArdeshir K.Analysis of Bombay Bazar [*sic*] VegetablesFood and Drugs119108789 BasuNarendra NathAnalysis of Jack Fruit SeedsFood and Drugs119108990

